# Machine Learning Models for Image-Based Diagnosis and Prognosis of COVID-19: Systematic Review

**DOI:** 10.2196/25181

**Published:** 2021-04-23

**Authors:** Mahdieh Montazeri, Roxana ZahediNasab, Ali Farahani, Hadis Mohseni, Fahimeh Ghasemian

**Affiliations:** 1 Medical Informatics Research Center, Institute for Futures Studies in Health, Kerman University of Medical Sciences Kerman Iran; 2 Computer Engineering Department, Faculty of Engineering Shahid Bahonar University of Kerman Kerman Iran

**Keywords:** machine learning, diagnosis, prognosis, COVID-19

## Abstract

**Background:**

Accurate and timely diagnosis and effective prognosis of the disease is important to provide the best possible care for patients with COVID-19 and reduce the burden on the health care system. Machine learning methods can play a vital role in the diagnosis of COVID-19 by processing chest x-ray images.

**Objective:**

The aim of this study is to summarize information on the use of intelligent models for the diagnosis and prognosis of COVID-19 to help with early and timely diagnosis, minimize prolonged diagnosis, and improve overall health care.

**Methods:**

A systematic search of databases, including PubMed, Web of Science, IEEE, ProQuest, Scopus, bioRxiv, and medRxiv, was performed for COVID-19–related studies published up to May 24, 2020. This study was performed in accordance with the PRISMA (Preferred Reporting Items for Systematic Reviews and Meta-analyses) guidelines. All original research articles describing the application of image processing for the prediction and diagnosis of COVID-19 were considered in the analysis. Two reviewers independently assessed the published papers to determine eligibility for inclusion in the analysis. Risk of bias was evaluated using the Prediction Model Risk of Bias Assessment Tool.

**Results:**

Of the 629 articles retrieved, 44 articles were included. We identified 4 prognosis models for calculating prediction of disease severity and estimation of confinement time for individual patients, and 40 diagnostic models for detecting COVID-19 from normal or other pneumonias. Most included studies used deep learning methods based on convolutional neural networks, which have been widely used as a classification algorithm. The most frequently reported predictors of prognosis in patients with COVID-19 included age, computed tomography data, gender, comorbidities, symptoms, and laboratory findings. Deep convolutional neural networks obtained better results compared with non–neural network–based methods. Moreover, all of the models were found to be at high risk of bias due to the lack of information about the study population, intended groups, and inappropriate reporting.

**Conclusions:**

Machine learning models used for the diagnosis and prognosis of COVID-19 showed excellent discriminative performance. However, these models were at high risk of bias, because of various reasons such as inadequate information about study participants, randomization process, and the lack of external validation, which may have resulted in the optimistic reporting of these models. Hence, our findings do not recommend any of the current models to be used in practice for the diagnosis and prognosis of COVID-19.

## Introduction

Since the COVID-19 outbreak was first reported in December 2019 in Wuhan, China, the number of people infected worldwide has exceeded 33 million (as of September 28, 2020) [1]. The World Health Organization declared COVID-19 as a global health emergency that requires international cooperation [2,3]. Despite many efforts to control the spread of the disease, many countries are facing a crisis of intensive care [4,5]. In order to reduce the burden on the health care system and provide the best possible care for patients, accurate and timely diagnosis and effective prognosis of COVID-19 is important and necessary. Moreover, early diagnosis of the disease helps health care providers prevent delays in providing the best possible treatment.

The diagnostic method currently used for COVID-19 is a positive result of a nucleic acid test such as real-time reverse transcription–polymerase chain reaction (RT-PCR) or next-generation sequencing [6]. Despite the advantages of this method, the number of false-negative test results due to unstable specimen processing is relatively high in clinical practice, which makes COVID-19 diagnosis difficult [7,8]. Moreover, laboratory testing for COVID-19 requires a rigorous platform, which is not assembled in all hospitals. Thus, COVID-19 testing may involve transfer of clinical specimens, which may delay diagnosis for days. Computed tomography (CT) plays a fundamental role in the diagnosis of disease progression, because of its excellent diagnostic accuracy and clinical outcomes [9]. For instance, lung CT images can be used to detect characteristic abnormalities associated with COVID-19 [10,11]. Characteristic imaging manifestations of COVID-19, such as ground-glass opacities, bilateral involvement, and peripheral distribution, have been described in various studies [12,13]. Consolidation, cavitation, and interlobular septal thickening imaging features have also been reported in some patients with COVID-19 [14,15].

Machine learning techniques have achieved considerable success in the field of medical imaging and image analysis owing to the use of deep learning technologies that allow for improved feature extraction [16,17]. Machine learning is a popular method of data analytics that uses different learning algorithms to teach computers to learn from data for performing related tasks. It is principally based on the learning methods and can be divided into three groups, namely, supervised (classification, regression, and ensembling), unsupervised (**association, clustering,** and **dimensionality reduction),** and reinforcement learning, with each category consisting of various methods for specific aims, such as instance-based algorithm, regression analysis, regularization, and classifiers for particular aims. Numerous studies have suggested the use of machine learning techniques in the diagnosis of diseases. For example, some studies have used deep learning techniques to diagnose and differentiate between bacterial and viral pneumonia using pediatric chest radiographic images [18,19]. Considerable effort has also been invested in diagnosing various chest CT imaging features that are characteristic of different diseases [20,21]. Various models ranging from rule-based systems to advanced machine learning models (deep learning) have been published in the context of the diagnosis and prognosis of COVID-19, which have substantially contributed to the field of health care by aiding the diagnosis and treatment of this disease and helped saved lives [22].

The objective of this systematic review was to identify publications in the existing literature that have used image processing methods based on CT images for the diagnosis and prognosis of COVID-19. We believe that this review would aid clinical practice by informing future research and development about improved diagnostic and treatment techniques for patients with COVID-19.

## Methods

### Information Source and Search Strategy

We conducted a systematic search of the databases, including PubMed, Web of Science, IEEE, ProQuest, Scopus, bioRxiv, and medRxiv, for articles published up to May 24, 2020. The study was performed according to the PRISMA (Preferred Reporting Items for Systematic Reviews and Meta-analyses) guidelines [23]. We used two groups of keywords for searching these databases—keywords related to the novel coronavirus and those related to machine learning and image processing. 

### Inclusion and Exclusion Criteria

All studies that applied image processing techniques for the prediction and diagnosis of COVID-19 were considered. We included original research articles regardless of the language of publication. We excluded editorials, commentaries, letters, books, presentations, conference papers, and papers without full text or those with insufficient information. To prevent duplication in data collection, we also excluded all types of review articles.

### Study Selection

**Figure 1:**
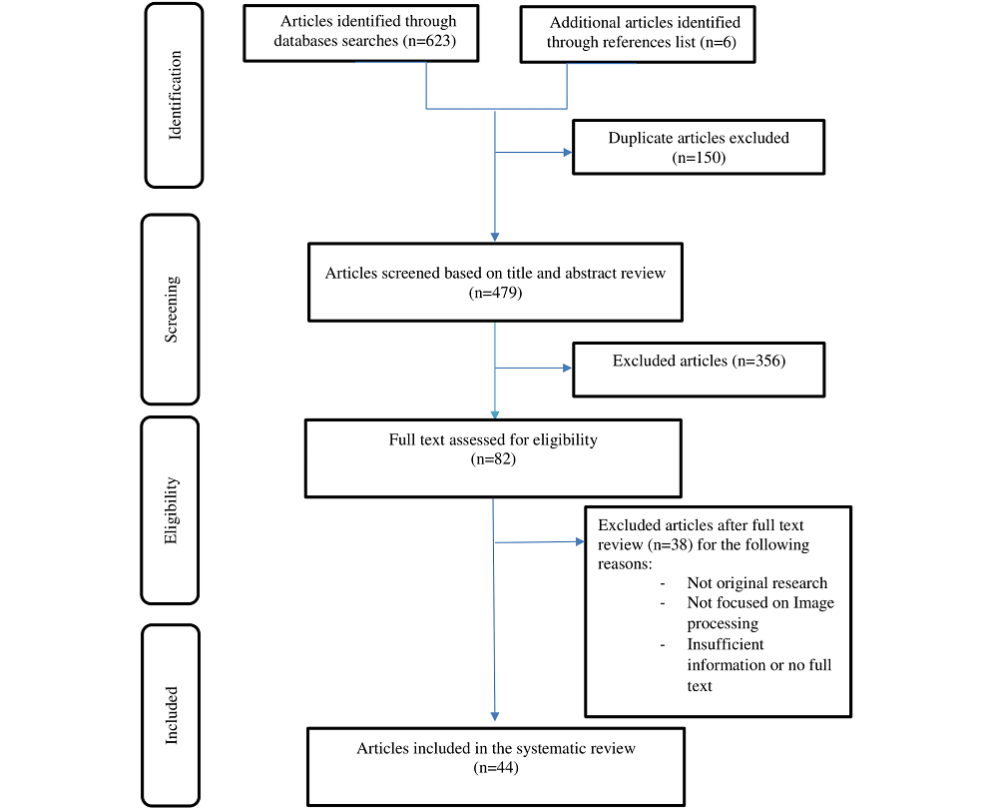
Study identification and selection process.

The selection process was initiated by removing duplicated articles. Thereafter, two reviewers worked independently to screen the titles and abstracts of the selected articles against the eligibility criteria. We further excluded articles that did not apply image processing for the prediction and diagnosis of COVID-19. The detailed process regarding the selection of articles is presented in Figure 1. After the initial screening, the same authors independently reviewed the full text of the relevant articles. Any disagreements were resolved through mutual discussion. During the screening of the articles, the reviewers documented the reasons for the exclusion of each article. We used a free web and mobile application platform (Rayyan, Qatar Computing Research Institute) for the screening of articles [24].

### Data Extraction and Synthesis

A standard data extraction form based on the Critical Appraisal and Data Extraction for Systematic Reviews of Prediction Modeling Studies (CHARMS) checklist was used by five reviewers [25]. A data extraction form was used to extract specific details about each article. This form consisted of information on imaging modality, database, scope, setting, data source and outcome, sample size (including training, validation, and testing), machine learning technique, performance, validation type, risk of bias (Multimedia Appendix 1). We investigated several forms of validation, for example, external (ie, evaluation in an independent database) and internal validation (ie, bootstrap validation, cross validation, random training test splits, and temporal splits).

### Risk of Bias Assessment

The risk of bias was assessed using the Prediction Model Risk of Bias Assessment Tool (PROBAST) [26].

## Results

### Overview

We retrieved 623 relevant studies through database searches. Six studies were identified from the reference lists of the selected publications. After title and abstract screening, 82 articles were selected for full-text assessment, which led to the exclusion of 38 articles due to various reasons.

In total, 44 studies were included in this systematic review (Figure 1). All included studies documented that patients’ CT and chest x-ray (CXR) images were processed for segmentation and classification tasks to enable the diagnosis and prognosis of COVID-19. These studies described a total of 89 deep learning and machine learning models applied for COVID-19 screening of CT and CXR images (Table 1).

**Table 1:** Deep learning architecture and parameters.

Study	Network architecture	Optimizer	Learning rate	Batch size
[27]	U-Net	—^a^	—	—
[28]	Efficient Net B4+2 FC [29]	SGD^b^	1e-4	64
[30]	ResNet-50-2D [31]	—	—	—
[32]	CPM^c^-Nets [33]	—	—	—
[34]	U-Net (segmentation)	—	1e-5	32
[34]	ResNet 152 (classification)	—	1e-5	32
[35]	U-Net	—	—	—
[36]	AlexNet, GoogLeNet, and ResNet-18 + GAN^d^	SGD	0.01	64
[37]	AlexNet, VGG-16, VGG-19, SqueezeNet, GoogLeNet, MobileNet-V2, ResNet-18, ResNet-50, ResNet-101, and Xception	SGD	0.01	—
[38]	50×5 layers + 8FC^e^ + 1 global average pooling + softmax 5 layers = (2 Conv + 3MP)	Adam	Optimize beside L2 regularization and momentum	32
[39]	VGG-19	Adam	0.001	15
[40]	DenseNet-201 + Inception_resnet_V2 + Inception_V3 + Mobilenet_V2 + ResNet-50 + VGG16 + VGG19 +	Adam	1e-5	32
[41]	2D (U-net + DRUNET + FCN^f^ + SegNet + DeepLabv3)	SGD	0.01	4
[41]	3D (ResNet-18)	Adam	0.001	8
[42]	CNN^g^ network base on the modification of ResNet-50 architecture	Rmsprop	1e-5	4
[43]	DenseNet like structure [44]	—	—	—
[45]	Model A, 22 layers	Adam	0.001	—
[45]	Model B, 28 layers	Adam	0.001	—
[45]	Model C, 29 layers	Adam	0.001	—
[46]	TB detection DL^h^ model	—	—	—
[47]	MobileNetV2, SqueezeNet	SGD	1e-5	64
[48]	Darknet-19	Adam	3e-3	—
[49]	2D (ResNet-50)	—	—	—
[49]	3D (U-Net)	—	—	—
[50]	ResNet-18	Adam	0.001	16
[51]	MobileNetV2	—	—	—
[52]	DenseNet	SGD	—	32
[53]	GAN + VGG16	Adam	0.001	16
[54]	U-Net	Adam	1e-4	—
[55]	FC-DenseNet-103	Adam	1e-4	2
[55]	ResNet-18	Adam	1e-5	16
[56]	DeCoVNet	Adam	1e-5	32
[57]	3D-ResNet (prediction)	Momentum	1e-4	—
[57]	3D-UNet (segmentation)	Momentum	1e-4	—
[58]	ConvNet [59]	Adam	1e-4	64
[60]	INF-Net	Adam	1e-4	16
[60]	FCN8s	SGD	1e-10	16
[61]	UNet++ [62]	—	—	—
[63]	FCN-8s, U-Net, V-Net, and 3D U-Net++	Adam	1e-4	—
[64]	VB-Net	—	—	—
[65]	VB-Net	—	—	—
[66]	M-Inception (6Conv + 3MP + inception + softmax + 2FC)	—	—	—
[67]	VNET_IR_RPN [68]	—	—	—
[69]	DRE-NET (ResNet-50 as the backbone)	—	—	—
[70]	U-Net as segmentation	Adam	1e-5	1
[70]	DeconvNet as prediction	Adam	1e-5	1
[71]	MLP^i^ + LSTM^j^ (single layer) + FC + softmax	—	—	—
[72]	U-Net	—	—	—

^a^Not available.

^b^SGD: stochastic gradient descent.

^c^CPM: cross partial multiview networks.

^d^GAN: generative adversarial network.

^e^FC: fully connected layer.

^f^FCN: fully convolutional network.

^g^CNN: convolutional neural network.

^h^DL: deep learning.

^i^MLP: multilayer perceptron.

^j^LSTM: long short-term memory.

### Dataset

Distribution of the 44 collected datasets showed that 12 (27%) studies used data on patients with COVID-19 from China; 3 (7%) studies used data on patients from China and USA [27,28,30]; 1 (2%) study used data on patients from China and Japan [32]; 1 (2%) study used data from China, USA, and Switzerland [34]; and 1 (2%) study used data from Italy [73], the Netherlands [35], and Canada [36]. Moreover, 11 (25%) studies were based on international data. Finally, the datasets used in 25 (56%) studies are publicly available, whereas those used in the rest of the studies (19/44, 43%) are nonpublic. The duration of follow-up was unclear for most studies. Only 2 (4%) studies reported follow-up time; the first one reported a follow-up of more than 5 days [28] and the other reported a follow-up of 3-6 days [37].

We categorized the reviewed studies (N=44) into three broad categories: (1) the *CT scan* category comprised 28 (63%) studies in which the models used chest CT images for abnormality analysis and COVID-19 diagnosis; (2) the *x-ray* category consisted of 14 (32%) studies in which the models use patients’ CXR images; and (3) the *hybrid* category consisted of 3 (7%) studies in which the models use a combination of CT, CXR, lung ultrasound, and other information such as the patient’s age and medical history. 

### Machine Learning Methods

Several machine learning techniques have been used for COVID-19 detection, prediction, and diagnosis. For the classification algorithms, the dataset is divided into training and test datasets. The model was developed using the training dataset, following which the validation of the training model was accomplished using the test dataset. For the segmentation algorithm, most studies used deep learning methods based on convolutional neural networks (CNNs) that have been used widely as a classification algorithm. In all, 40 studies used diagnostic models, whereas 4 studies used prognostic models for patients who had received a COVID-19 diagnosis [41,43,71,72]. Table 1 illustrates the deep learning architectures and hyperparameters used in the included studies using deep learning methods. In this table, the three most important parameters such as optimizer method, learning rate, and mini-batch size were considered. In the case of the optimizing algorithm Adam and RMSProp, all reported learning rates are initial values except in one study [29] that used a constant learning rate value.

### Diagnostic Models to Detect COVID-19 in Patients With Suspected Infection

For better categorization among the various machine learning methods used in the studies analyzed, we classified the models into two groups: CNN-based models (n=31) and other machine learning algorithms (n=8). Among these, 31 studies used 61 CNN-based algorithms, which were further subdivided as follows: U-Net (n=10), ResNet (n=11), SqueezeNet (n=3), MobileNet (n=4), multiple types of VGG networks (n=4), GoogLeNet (n=2), and others (n=4). A total of 8 studies used 26 other machine learning methods, of which support vector machine (SVM) was the most commonly used algorithm as a classifier (n=5) [32,73-76], followed by random forest (n=1) [65,76], logistic regression (n=1) [34], and other machine learning algorithms (n=3). In addition, 1 study [77] used a multi-objective, differential, evolution-based algorithm to automatically build CNN. In addition, 4 models were developed and externally validated in the same study (in an independent dataset, excluding random training test splits and temporal splits) [28,30,46,55].

### Prognostic Models for Patients With a COVID-19 Diagnosis

**Figure 2:**
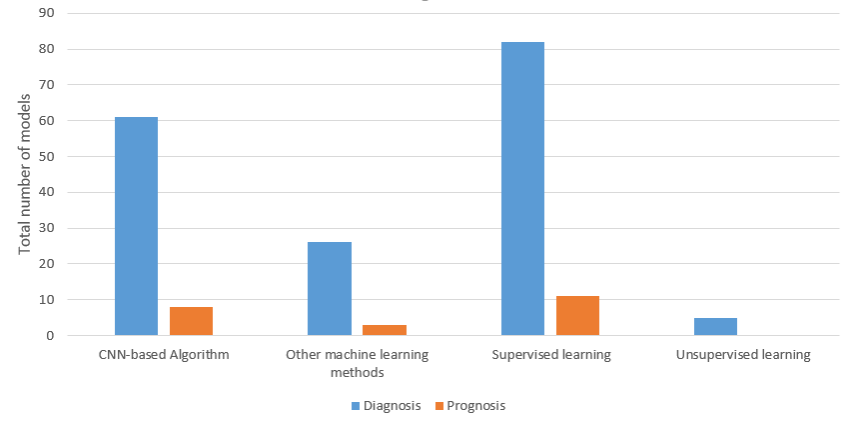
Number of deep learning and other machine learning methods used in the reviewed studies. CNN: convolutional neural network.

We identified 4 prognostic models for patients who had received a COVID-19 diagnosis. One of these models used a CNN-based model to estimate mortality risk in patients with suspected or confirmed COVID-19 and externally validate using another dataset [43]. Two models aimed to predict disease progression to a severe or critical state, and one of these two models used five CNN-based algorithms [41]. The fourth prognostic model used an LSTM network and compared it with other traditional methods such as principal component analysis, linear discriminant analysis, SVM, and multilayer perceptron [71]. Furthermore, 1 study [72] aimed to develop a random forest algorithm and a logistic regression model to predict the length of hospital stay (greater than 10 days) and estimated C indices of 0.92 and 0.96, respectively. The other studies did not report the C index. Figure 2 shows the bar graph for all methods used in the included studies.

In our analysis, we found that almost all studies had problems with the lack of sufficient data. To address this problem, some studies used data augmentation to synthesize new data, some others attempted to use a combination of different datasets or different kinds of data in their study, and other studies tried to take advantages of non–neural network–based methods such as k-nearest neighbor, SVM, and feature extraction methods. In general, studies that used deep CNNs produced better results than those using non–neural network–based methods. Moreover, 18 studies used K-fold cross-validation, whereas 19 of them used random training test split as a validation method.

### Risk of Bias

According to the PROBAST assessment tool [26], all included studies were at a high risk of bias, which suggests that their predictive performance when used in practice is probably lower than that reported. Most of the studies were at high risk in the participant domain due to the lack of information about patients and intervention groups. Moreover, almost all studies obtained a high index in the analysis domain, which shows that most of the deep learning models did not have interpretability and that the results were probably lower than those obtained using real datasets.

As shown in Table 2, 15 of the 44 (34%) studies had a high risk of bias for the participant domain, which indicates that these articles did not contain adequate information about the enrolled study participants and intervention groups. In addition, any imbalances in the datasets could cause problems in the randomization process (eg, imbalances between the number of images of normal cases and COVID-19 or other pneumonia cases), leading the study to a risk of bias. Unclear reporting on the inclusion of participants prohibited a risk of bias assessment in 15 (34%) studies. On the other hand, 19 (43%) studies had a high risk of bias due to the predictor domain; this may be attributed to the high false-negative ratio of COVID-19 diagnostic tests (eg, RT-PCR) due to which CT and x-ray images may be wrongly classified as COVID-19, thus leading to inaccurate learning of the models and missing outcome data to predicting processes. In addition, an unclear index was reported in 13 (30%) articles, implying that these articles did not provide specific information about the missing outcome data.

**Table 2:** Risk of bias assessment (using Prediction Model Risk of Bias Assessment Tool) based on four domains conducted for all studies included in the review.

Study	Domain					Overall risk of bias
	Participants	Predictors	Outcome	Analysis		
[27]	Unclear	Low	High	Unclear	High	
[28]	High	High	High	High	High	
[30]	Unclear	Unclear	High	High	High	
[32]	Unclear	High	High	High	High	
[34]	High	Unclear	Unclear	High	High	
[73]	Unclear	Unclear	Unclear	High	High	
[35]	High	High	High	High	High	
[36]	High	High	Low	Unclear	High	
[37]	Low	Low	Unclear	Unclear	Unclear	
[38]	Unclear	High	Low	High	High	
[39]	High	Unclear	Unclear	High	Unclear	
[40]	Unclear	Low	Low	High	High	
[41]	Low	Low	Low	High	High	
[42]	Some concern	High	Low	Unclear	High	
[76]	High	High	High	High	High	
[43]	Unclear	Low	High	Unclear	High	
[45]	High	High	Low	High	High	
[46]	High	High	High	High	High	
[75]	Unclear	High	Unclear	High	High	
[74]	Unclear	Low	Unclear	Unclear	Unclear	
[47]	Unclear	Low	High	Unclear	High	
[48]	Unclear	Low	Unclear	High	High	
[49]	Low	Unclear	Low	High	High	
[77]	Unclear	High	High	High	High	
[50]	Low	High	High	High	High	
[51]	Unclear	High	High	High	High	
[52]	High	High	High	High	High	
[53]	High	High	Low	High	High	
[54]	High	High	High	High	High	
[55]	High	High	Low	High	High	
[56]	Low	Low	Unclear	High	High	
[57]	Unclear	Low	High	High	High	
[58]	High	High	High	High	High	
[60]	High	High	High	High	High	
[61]	High	Unclear	Low	High	High	
[63]	High	Unclear	High	High	High	
[64]	Unclear	Unclear	High	High	High	
[65]	High	Unclear	Low	High	High	
[66]	High	Unclear	Low	High	High	
[67]	High	Unclear	High	High	High	
[69]	Unclear	Unclear	Low	High	High	
[70]	Unclear	Unclear	High	High	High	
[71]	Low	Unclear	Unclear	High	High	
[72]	Unclear	Unclear	Low	Low	High	

Published research articles often do not provide clear information about the preprocessing steps, such as cropping of images. Furthermore, due to the complexity of the machine learning algorithms used to process images into predictors, it is challenging to fully apply the PROBAST predictors. Most models were at high risk of bias in the outcome domain because most of the studies used inappropriate measurement, or there was no reason that the measurement or ascertainment of the outcome differed among intervention groups. Finally, none of the models were identified to be at low risk of bias in the analysis domain. Although many datasets have been made available to researchers in recent months to diagnose COVID- 19, there remains a lack of training data, which increases the risk of overfitting. Five models were developed and externally validated in the same study (in an independent dataset, excluding random training test splits and temporal splits).

### Metrics

For a more comprehensive review, we classified machine learning–based COVID-19 diagnostic techniques into three major categories based on the imaging modality used in the study. In the following sections, we discuss each category in detail.

#### CT Scan Category

all machine learning methods that were classified in the CT category used CT scan images in their analyses. Since CT scan data have a 3D nature, two approaches were generally followed. The first is a slice-based approach in which each slice of a CT scan image is analyzed independently; then, at the stage of decision-making, voting is used to decide whether the CT scan image belongs to COVID-19–positive class or COVID-19–negative class. In the second approach, all slices of a CT scan were used as a 3D-like set and used in a 3D CNN [45,57]. The investigations showed that methods utilizing a slice-based approach have a better performance in terms of COVID-19 diagnosis.

For example, Pu et al [45] proposed three 3D CNN models to classify pneumonia and COVID-19 cases by using CT scans. They analyzed 498 CT scans of patients with COVID-19 and 497 CT scans of patients with pneumonia in their experiments. Thus, 256 slices of each CT scan were used as input to the models. Although the results showed that the model with a higher number of layers had the best performance with an area under the curve (AUC) of 0.7, their model could not distinguish between pneumonia and COVID-19 well enough.

Among the methods utilizing a slice-based approach, the proposed method by Ardakani

et al [37] reported the best performance with an accuracy of 0.99 and a sensitivity of 1.0. They trained 10 different well-known CNNs by using 1020 slices of 108 CT scans to distinguish COVID-19 from other pneumonias and normal cases. ResNet-101 demonstrated the best sensitivity and was reported as an efficient model for COVID-19 diagnosis by using CT images. Although ResNet-101 had the best sensitivity, it had the weakest results in terms of specificity as compared to Xception and ResNet-50 models, which implies that ResNet-101 might be involved in overfitting.

Some other studies [28,41,56] also reported an accuracy higher than 0.96. The common factor in these approaches was the high level of augmentation used. For instance, Zhang et al [41] used 4695 CT slices that was increased to more than 600,000 slices by using augmentation techniques. Owing to the significance of the number of available images in the training of deep CNN models, some studies attempted to use non–CNN-based methods such as feature extraction, thresholding, and transformation-based methods.

As an example, Fang et al [74] used a radiometric feature extraction technique for all slices of available CT scans (including CT scans of 46 COVID-19–positive and 26 other pneumonia cases); the extracted features were used to train an SVM classifier for further classification. In the test phase, their method achieved an AUC of 0.76. Because other measurements such as accuracy and sensitivity were not reported [74], high risk of bias is very probable.

Due to the difference in color and texture of healthy and infected regions in the lung images, some researchers tried to exploit texture information in their studies. For example, El Asnaoui et al [40] used different feature descriptors such as local binary pattern, gray level co-occurrence matrix, and discrete wavelet transform to analyze local features in images. Finally, in the decision-making stage, an SVM classifier was used to determine whether an input image belongs to the COVID-19 class or not. The results show that this method could achieve a sensitivity of 0.93 and a specificity of 1.0.

#### X-ray Category

Although a CT scan generates high-quality images with more details than an x-ray image, some studies have attempted to use x-ray images to investigate the probability of COVID-19 diagnosis. Among the studies we reviewed, 14 studies used CXR images in their analyses. Yi et al [46] proposed a hypothesis that a deep CNN model trained on a similar dataset can be useful in COVID-19 diagnosis. They trained a ResNet model for pulmonary tuberculosis (TB) detection by using CXR images from the NIH Chest X-ray dataset [78], which did not have any information of TB, yet the trained model achieved a high performance with regard to TB detection. The same approach had been used for COVID-19 diagnosis, and the x-ray images of 88 COVID-19–positive patients were inputted into the trained model. The results showed that the model could correctly classify 78 of the 88 (89%) input x-ray images and that it misclassified 10 input x-ray images. Although the reported results are satisfactory, they did not consider COVID-19–negative inputs and did not measure the false-positive rates of the proposed methods.

A continuously growing dataset has been provided by a group of researchers at the University of Montreal [79], which includes annotated CXR images of patients with COVID-19. Several studies [39,40,47,48,51,55] have used this dataset in their analyses. For instance, Han et al [55] proposed a DenseNet model with a relatively small number of parameters and used a combination of x-ray images from various datasets, including the COVID Chest X-Ray dataset (180 COVID-19–positive images), JSRT (20 normal images), NLM (73 normal and 57 tuberculosis images), and CoronaHack (98 normal and 54 pneumonia images), for the training and testing phases. The trained model achieved an accuracy of 0.88 and a precision of 0.83.

Another study [27] utilized images from a pneumonia dataset, including 22,000 CXR images, to train a U-Net model to compute the probability of pneumonia using x-ray images at the pixel level. By integrating the probability values of pixels as a single image, a class activation map is obtained that can be used to show which region in the input image has the most relevance to pneumonia. After model training, they fed 10 CXR images from 5 patients that were captured on several consecutive days. They reported that their model could detect localized areas of pneumonia with increasing likelihood as the subtle airspace opacities increased over time. However, no technical information and measurements were described.

Some other studies [35,48,55] also used a class activation map to not only classify each image into COVID-19–positive and COVID-19–negative classes but also to localize suspected areas in CXR images.

#### Hybrid Category

Given that most of the included articles mentioned data shortage as a major problem in developing an efficient COVID-19 diagnosis model, some studies tried to exploit two or more types of data in their analyses. For instance, in the study by Wang et al [43], at the first stage, a CNN model was trained on 4106 CT slices with epidermal growth factor receptor data. In the second stage, 709 COVID-19–positive images from patients from Wuhan city were used to retrain the model. Finally, 458 images from four different cities in China were used as test images, and the model achieved an accuracy of 0.85 and a sensitivity of 0.80.

In the study by Mei et al [50], clinical data such as patient’s age, gender, symptoms, and laboratory findings were used in addition to CT scans of 905 patients with suspected COVID-19 from 13 provinces in China. A modified ResNet model was proposed by the authors to accept clinical data alongside the CT scan slice images. The results showed that their proposed model achieved an accuracy equivalent to a senior chest radiologist with an AUC of 0.86. Although their dataset is not publicly available, the trained models are available for others to download.

## Discussion

### Principal Findings

In this study, we reviewed 44 studies related to the diagnosis and prognosis of COVID-19 that used advanced machine learning techniques based on clinical images to diagnose COVID-19 or COVID-19–related pneumonia, or to assist with the segmentation of lung images by using chest CT and x-ray images with their proposed machine learning methods. The predictive performance measures showed a high to almost perfect ability to detect COVID-19. Overall, 24 different methods, such as deep CNNs, local feature descriptors, and decision trees, were used in the reviewed studies; however, some of them used similar models with a different setup or configuration.

Due to the complexity of the clinical images used and the need to obtain the best results for an early diagnosis of COVID-19, most of the reviewed articles (36/44, 82%) had based their learning algorithm on neural networks and deep learning as proven, powerful learning methods. However, deep CNNs, which are developed in principle to work with images, require sufficient amount of data for fine-tuning the network parameters.

Given that the COVID-19 outbreak was in the early stage at the time of this review and that there was a lack of proper data available, most of these CNN-based studies were endangered by overfitting, which causes a high risk of bias. Nevertheless, some of the studies used previously available data of chest CT or x-ray images to compensate with data shortage and to enrich the training data. For instance, Ucar and Korkmaz [38] used 66 COVID-19–positive lung x-ray images, which were not sufficient to train a CNN. To overcome this problem, they added these images to the images of a publicly available pneumonia dataset called Chest X-Ray Images (Pneumonia) [80], which was used to obtain access to a larger number of images for network training. Although the pneumonia dataset does not provide any information about COVID-19, it can enhance the model performance to better distinguish between healthy and unhealthy lungs. Another approach used for compensating the lack of data was to utilize data augmentation techniques such as image mirroring and blending. Although most of the reviewed studies used simple augmentation methods, some used more complicated techniques. For example, in the study by Ucar and Korkmaz [38], a generative adversarial network was trained to synthesize new images from the limited 307 images available that were not considered enough for network training.

This systematic review is in its early stage, and we will continue to update our findings and evaluation to provide new information to health care professionals and decision makers as more international studies are conducted over time.

### Study Limitations

With the rapid publication of COVID-19 prediction models in the medical image processing domain in the recent past, this systematic review cannot be considered as an up-to-date list of all the current prediction models.

### Conclusions

Different models have been proposed for the diagnosis and prognosis of COVID-19, demonstrating varying levels of discriminative performance. The results show that deep CNNs dedicated a larger number of models than non–neural network–based methods; moreover, deep networks achieved better results than other machine learning models. However, the rapid spread of COVID-19 and the lack of data for machine learning approaches and training may have increased the likelihood of overfitting and vague reporting. Furthermore, the lack of adequate information about patients and study participants likely led to the high risk of bias, which made the results seem optimistic. Therefore, the performance of these models is misleading, and we do not recommend their practical use. Future studies aimed at using deep neural networks for diagnosing COVID-19 should address aspects of appropriate model performance by using a larger training dataset with no imbalance and complete information about patients and intervention groups.

## Appendix

Multimedia Appendix 1 Table S1. Characteristics of included articles.

## Abbreviations

AUC: area under the curve
CNN: convolutional neural network
CT: computed tomography
CXR: chest x-ray
GAN: generative adversarial network
PROBAST: Prediction Model Risk of Bias Assessment Tool
RT-PCR: reverse transcription–polymerase chain reaction
SVM: support vector machine
