# Supplementary material for: Machine Learning Models for Image-Based Diagnosis and Prognosis of COVID-19: Systematic Review
Source: JMIR Med Inform. 2021 Apr 23;9(4):e25181. doi: 10.2196/25181 (PMC8074953; doi:10.2196/25181)
Supplement: Multimedia Appendix 1 [file medinform_v9i4e25181_app1.pdf]

Article	Imaging modality	Database	Scope	Setting, data source and outcome	Sample Size(train, (validation) ,test)	Machine learning technique	Performance (best) (%)	Validation type	ROB
[27]	Chest X-ray	-	Segmentation	early diagnosis and longitudinal follow-up of suspected pneumonia	22k(unclear)	Deep learning	-	unclear	High
[28]	CT	-	Classification and segmentation	evaluate an artificial intelligence (AI) system in differentiating COVID-19 and other pneumonia on chest CT and assess radiologist performance without and with AI assistance	32583 (830,237,119)	Deep learning(CNN)	Accuracy: 96<br>Sensitivity: 95<br>Specificity: 96	External	High
[30]	CT	data from China and US	Classification	present a system that utilizes robust 2D and 3D deep learning models	50 patient (unclear)	Deep learning	C index 99 (98 to 100)	External	High
[32]	CT	Tongji Hospital of Huazhong University	Classification and segmentation	propose to conduct diagnosis of COVID-19 with multi-view representation learning	2522 (70%, 30%))	SVM, Logistic-Regression (LR), Gaussian- Naive-Bayes (GNB), K-Nearest-Neighbors (KNN), And Fully-Connected-Neural Networks (NN)	Accuracy: 93<br>Sensitivity: 100<br>Specificity: 91	5-fold cross validation	High
[34]	CT	data from China, US, and Switzer-land	Classification	propose an artificial intelligence (AI) system for fast COVID-19 diagnosis	416 patient (220,196)	Deep learning	C index 98,<br>Sensitivity: 94,<br>Specificity: 95	Training test split	High
[73]	CT	data from Italy	Classification	early phase detection of Coronavirus (COVID-19) by machine learning methods	53 (unclear)	GLCM, LDP, GLRLM, GLSZM, DWT and SVM	Sensitivity: 93,<br>Specificity: 100	Cross validation	High
[35]	CXR	Nonpublic+kgg dataset	Classification	evaluated an AI system for detection of COVID-19 characteristics on frontal chest radiographs	24678 (23138,1540, 468)	Deep learning	AUC: 81<br>Sensitivity:85<br>Specificity: 65	Training test split	High
[36]	CXR	Dr.Joseph Cohen data+ dataset	Classification	introduced a transfer of deep learning models to classify COVID-19 X-ray images	307 (reach 8100 after GAN)	Deep learning	Accuracy: 100 Binary<br>Accuracy: 84-class Google Net has had	Training test split	High

Article	Imaging modality	Database	Scope	Setting, data source and outcome	Sample Size(train, (validation) ,test)	Machine learning technique	Performance (best) (%)	Validation type	ROB
		Chest X-ray Images			(80%, 20%)		the best performance for 4-class scenario -> Precision: 84, F1 Score:82 ,R e c a l l : 80		
[37]	CT	Iran University of medical science	Classification	propose a CAD system based on deep learning to classify COVID-19 infection versus other a typical and viral pneumonia dis- eases	1020 CT (80%, 20%)	Deep learning	Accuracy: 99 Sensitivity: 100 Specificity:99	Training test split	unclear
[38]	Chest X-ray	COVID Chest X-ray ,Kaggle and pneumonia	Classification	rapid diagnostic of the COVID-19 based on deep Bayes-Squeeze Net	5949 (80%, 10%, 10%)	Deep learning	Accuracy: 98, Specificity: 99, F1: 98	Training test split	High
[39]	Chest X-ray	Chest X-ray scans	Classification	developed a CNN-based model to perform COVID-19 detections from chest radiographers	108k (80%, 10%, 10%)	Deep learning	Accuracy: 96 ,Precision: 91, Recall: 97	Training test split	High
[40]	X-ray and CT	chest X-ray and CT dataset	Multiclass classification	training and classification on several deep model	6087 (80%, 20%)	Deep learning	Accuracy: 92 Sensitivity: 92 Specificity: 96 Precision: 92 F1:0.92	Training test split	High
[41]	CT	CC-CCII	Segmentation and Classification	developed an AI system that can diagnose NCP and differentiate it from other common pneumonia and normal controls	617775 (80%, 10%, 10%)	Deep learning and Decision tree(GBDT)	Accuracy: 96 , Dice: 95	5-fold cross validation	High
[42]	CT	-	Classification	trained a multi-view fusion model using deep learning network to screen patients with COVID-19 using CT image	495 (395,50,50)	Deep learning	Accuracy: 76 Sensitivity:81 Specicificity:61	Training test split	High

Article	Imaging modality	Database	Scope	Setting, data source and outcome	Sample Size(train, (validation) ,test)	Machine learning technique	Performance (best) (%)	Validation type	ROB
[76]	X-ray	COVID-19, SARS, Pneumocystis and Streptococcus Images Varicella And MERS images	Classification	novel proposed hierarchical classification approach	3049 (2696,353)	K-Nearest Neighbors (KNN); Support Vectors Machine (SVM); Multilayer Perceptron (MLP); Decision Trees (DT); and Random Forests (RF)	F1 Score: 89	5-fold cross validation	High
[43]	CT	COVID-19 dataset + CT-EGFR	Classification and segmentation	proposed a novel fully automatic DL system using raw chest CT image to help COVID-19 diagnostic and prognostic analysis	4106 + 1266 (709,4 validation group(226, 161,53, 117)	Deep learning	Accuracy:85, Sensitivity:80, Specificity:89, F1Score:90	External	High
[45]	CT	Nonpublic dataset(COVID 19 and CAP)	Classification	proposed a novel fully automatic DL system using raw chest CT image to help COVID-19 diagnostic and prognostic analysis	497 + 498(CA) (490, 82, 77)	Deep learning	Sensitivity:84 Specificity:76 AUC: 70	Training test split	High
[46]	X-ray	NIH Chest X-Ray dataset	Classification	appropriately localized abnormalities for both focal and multi focal disease	11000+88(frontal CXR (unclear)	Deep learning	Accuracy: 89	External	High
[75]	X-ray	dataset from Kaggle and GitHub	Classification	presented an automated corona detection method by using novel feature generation model and an iterative feature selector	321 lung X-ray images (unclear)	Relief (IRF) based feature selection ,Decision tree linear discriminant (LD), support vector machine (SVM), k nearest neighbor- hood (KNN) and	Accuracy: 99 Sensitivity: 98 Specificity: 100	10-fold cross validation	High

Article	Imaging modality	Database	Scope	Setting, data source and outcome	Sample Size(train, (validation) ,test)	Machine learning technique	Performance (best) (%)	Validation type	ROB
						subspace discriminant (SD)			
[74]	CT	Beijing Youan hospital	Segmentation and clustering	lung lesion segmentation ,image preprocessing, feature extraction, feature selection and signature construction	75 Patients (50, 25)	SVM and Radiometric feature extraction	AUC: 73	3-fold cross validation	Unclear
[47]	CXR	GitHub source with MERS, SARS, COVID-19 and Qatar University	Classification and feature extraction + transfer learning	Preprocessed each image with deep learning models+ data reconstructed using the Fuzzy technique + training by deep learning models + classified by SVM	458 images (70%, 30%)	SVM and deep learning	Accuracy: 98<br>Sensitivity: 99<br>Specificity:99	5-fold cross validation	High
[48]	CXR	chest x-ray8 database	Classification	Deep learning model is proposed for the automatic diagnosis of COVID-19	1127 (80%, 20%)	Deep learning	Accuracy(Binary): 98<br>Accuracy: Multi:87<br>Sensitivity:85<br>Specificity: 92<br>Precision: 89<br>F1 Score: 87	5-fold cross validation	High
[49]	CTR	Nonpublic dataset	Classification	proposed a three-dimensional deep learning	4356 chest CT (90%, 10%)	Deep learning	AUC: 96<br>Sensitivity: 90<br>Specificity: 96<br>CAP -> AUC: 95<br>Sensitivity: 87<br>Specificity: 92 non-pneumonia -> AUC: 98 Sensitivity: 94<br>Specificity: 96	Training test split	High
[77]	CT	Chest CT images dataset	Classification	multi-objective differential evolution (MODE)–based convolutional neural net- works(CNN) for classification of COVID-19-infected patients from chest CT images	Unclear (Different validation ratio)	Deep learning	ACC: 1.9789,<br>F-measure:2.0928<br>Sensitivity: 1.8262<br>Specificity:1.6827<br>Kappa statistics:1.9276	20-fold cross validation	High

Article	Imaging modality	Database	Scope	Setting, data source and outcome	Sample Size(train, (validation) ,test)	Machine learning technique	Performance (best) (%)	Validation type	ROB
[50]	CT	Nonpublic dataset	Classification	Used artificial intelligence (AI) algorithms to rapidly diagnose patients who are positive for COVID-19	905 patients (80%, 20%)	Deep learning	Accuracy: 17.25<br>AUC: 92<br>Sensitivity: 84<br>Specificity: 82	Training test split	High
[51]	CXR	COVID-19 X-Ray	Classification	state-of-the-art Convolutional Neural Network called Mobile Net is employed and trained from scratch to investigate the importance of the extracted features for the classification task	3905 X-ray images (90%, 10%)	Deep learning	Accuracy: 91<br>Sensitivity: 97<br>Specificity: 99<br>Accuracy<br>7-class: 51	10-fold cross validation	High
[52]	CT	-	Classification + CAM	develop a DL model to evaluate the efficacy of Dense Net for detection of COVID-19 features on high resolution CT	295 (135,20,140)	Deep learning	AUC: 98<br>Accuracy: 92<br>Sensitivity: 97<br>Specificity: 87<br>F1 Score: 93	four-fold cross validation	High
[53]	CXR	COVID-19 radiography database kaggle dataset + data synthesis using GAN	Classification	Auxiliary Classifier (AC- GAN) COVID GAN for the generation of synthetic CXR images.+ Design a CNN-based Using COVID GAN for augmentation of training dataset	1124 (932, 192)	Deep learning	Accuracy: 95<br>Sensitivity: 90<br>Specificity: 97	Training test split	High
[54]	CT	TCIA dataset	Classification and segmentation	presented a novel weakly supervised deep learning framework that is capable of learning to detect and localize lesions on COVID- 19 and CAP CT scans from image-level label only	60 3D CT (40, 10, 10)	Deep learning	Accuracy: 90<br>AUC: 95<br>Sensitivity: 90<br>Specificity: 91	5-fold cross validation	High
[55]	CXR	JSRT Dataset + NLM dataset	Classification and segmentation	Propose a patch-based convolutional neural network approach with a relatively small number of trainable parameter for COVID-19 diagnosis.	247<br>JSRT 138<br>USNLM<br>(354, 49, 99)	Deep learning	Accuracy: 88<br>Precision: 83<br>Recall: 85<br>F1 Score: 84<br>Specificity:96	External	High

Article	Imaging modality	Database	Scope	Setting, data source and outcome	Sample Size(train, (validation) ,test)	Machine learning technique	Performance (best) (%)	Validation type	ROB
[56]	CTR	-	Classification	propose an attention based deep 3D multiple instance learning (AD3D- MIL)	460 chest CT (60%, 20%, 20%)	Deep learning	Binary:<br>Accuracy:97<br>AUC: 99<br>F1 Score: 97<br>Precision:97<br>Recall:97<br>Multi-Class:<br>Accuracy: 94<br>AUC: 98<br>F1 Score: 92<br>Precision:95<br>Recall:90	5-fold cross validation	High
[57]	CT	LUNA-16 dataset+ nonpublic dataset	Classification + CAM	lobe segmentation using 3D-Unet and)pneumonia prediction using 3D-ResNets with prior- attention mechanism	4597 (3997,600)	Deep learning	Accuracy: 93<br>Sensitivity: 87<br>Specificity: 95	5-fold cross validation	High
[58]	LUS (lung ultra-sonography)	Italian COVID-19 Lung Ultrasound Database (ICLUS-DB)	Classification and segmentation	present a novel fully-annotated dataset of LUS images collected from several Italian hospitals, with labels indicating the degree of disease severity at a frame-level, video level, and pixel-level (segmentation masks).	58924 Frames (70%, 30%)	Deep learning	Accuracy: 96<br>DICE:75<br>binary DICE:65<br>multi-class	5-fold cross validation	High
[60]	CT	100 labeled CT slices From the COVID-19 CT Segmentation dataset And 1600 unlabeled images from the	Classification and segmentation	Lung Infection Segmentation Deep Network (Inf-Net) is proposed to automatically identify infected regions from chest CT slices + semi-supervised segmentation framework based on a randomly selected propagation strategy,	1700 Labeled: (45, 5, 50)	Deep learning	MAE: 6.4	Training test split	High

Article	Imaging modality	Database	Scope	Setting, data source and outcome	Sample Size(train, (validation) ,test)	Machine learning technique	Performance (best) (%)	Validation type	ROB
		COVID-19 CT Collection dataset							
[61]	CT	data from China	Classification	construct a system based on deep learning for detecting COVID-19 pneumonia on high resolution CT	46096 anonymous images (40, 11)patients	Deep learning	Sensitivity:100 Specificity: 82	Training test split	High
[63]	CT	data from China	Classification	building and deploying an AI system that automatically analyzes CT images to detect COVID-19 pneumonia features	1136 patient (723, 259,154)	Deep learning	C index: 99, Sensitivity: 97 Specificity:92	Training test split	High
[64]	CT	data from China	Segmentation	a deep learning (DL)-based segmentation system is developed to automatically quantify infection regions of interest (ROIs) and their volumetric ratios w.r.t. the lung	249 patient (unclear)	Deep learning	Dice similarity coefficient 91.6	Training test split	High
[65]	CT	data from China	Classification and segmentation	All images were preprocessed to obtain the segmentations of both infections and lung fields, which were used to extract location-specific features	2685 patient (80%, 20%)	Random Forest	C index 94	5-fold cross validation	High
[66]	CT	data from China	Classification	using deep learning methods to extract COVID-19's specific graphical features and provide a clinical diagnosis	338 patient (259, 79)	Deep learning	C index 81 (71 to 84), Sensitivity: 83, Specificity 67	Internal, other image from same people	High
[67]	CT	data from China	Classification	establish an early screening model to distinguish COVID-19 pneumonia from Influenza-A viral pneumonia and healthy cases with pulmonary CT images using deep learning techniques	629 patient (509,90, 30)	Deep learning and Bayesian function	Sensitivity 87 PPV:81	Training test split	High

Article	Imaging modality	Database	Scope	Setting, data source and outcome	Sample Size(train, (validation) ,test)	Machine learning technique	Performance (best) (%)	Validation type	ROB
[69]	CT	data from China	Classification	develop an accurate computer-aided method to assist clinicians to identify COVID-19-infected patients by CT images	192 patient (131,34,27)	Deep learning	C index 96	Training test split	High
[70]	CT	data from China	Classification	Developing a deep learning-based model for automatic COVID-19 detection on chest CT	unclear	Deep learning	C index 96	Temporal validation	High
[71]	CT	data from China	Prediction	establish an AI model to predict mild patients with potential malignant progression	187 patients (133,54)	Deep learning and logistic regression	C index 95 (94 to 97)	5-fold cross validation	High
[72]	CT	data from China	Prediction	Intervention CT radiomics models based on logistic regression (LR) and random forest (RF) were developed on features extracted from pneumonia lesions in training and inter-validation datasets	31patients (26,5)	logistic regression (LR) and random forest (RF)	C index 92	5-fold cross validation	High
